# The Effect of Sub- and Near-Critical Carbon Dioxide Assisted Manufacturing on Medical Thermoplastic Polyurethane

**DOI:** 10.3390/polym15040822

**Published:** 2023-02-07

**Authors:** Sarn-ii Baru, Siobhan Matthews, Eric Marchese, Philip Walsh, Austin Coffey

**Affiliations:** 1 Convergent Technologies Research Group (CTRG), South East Technological University, X91 K0EK Waterford, Ireland; 2 SCF Processing Ltd., A92 AW74 Drogheda, Ireland; 3 Vascular Research and Development, Teleflex Inc., Wyomissing, PA 19610, USA; 4 Faculty of Engineering, Thammasat University, Pathum Thani 12120, Thailand

**Keywords:** supercritical carbon dioxide, hot melt extrusion process, polyurethane

## Abstract

Incorporating thermally labile active pharmaceutical ingredients for manufacturing multifunctional polymeric medical devices is restricted due to their tendency to degrade in the hot melt extrusion process. In this study, the potential of sub- and near-critical carbon dioxide (CO_2_) as a reversible plasticiser was explored by injecting it into a twin-screw hot melt extrusion process of Pellethane thermoplastic polyurethane to decrease its melt process temperature. Its morphological, throughput, thermal, rheological, and mechanical performances were also evaluated. The resultant extrudates were characterised using scanning electron microscopy, parallel plate rotational rheometer, differential scanning calorimetry, thermogravimetric analysis, and tensile testing. The process temperature decreased from 185 to 160 °C. The rheology indicated that the reduction in melt viscosity was from 690 Pa.s to 439 Pa.s (36%) and 414 Pa.s (40%) at 4.14 and 6.89 MPa, respectively. The tensile modulus in the elastomeric region is enhanced from 5.93 MPa, without CO_2_ to 7.71 MPa with CO_2_ at both 4.14 and 6.89 MPa. The results indicate that the employment of both sub- and near-critical CO_2_ as a processing aid is a viable addition to conventional hot melt extrusion and that they offer more opportunities for thermosensitive drugs to be more stable in the molten stream of Pellethane thermoplastic polyurethane.

## 1. Introduction

In recent years, polymers have been applied for several medical applications, such as medical devices, implants, and tissue engineering constructs. The wide range of different biomedical synthetic polymeric materials include silicone, Teflon, polyurethane (PU), polyethylene (PE), and polyvinyl chloride (PVC) [1,2,3,4,5]. Among these polymeric materials, polyurethane (PU) is one of the preferred polymers due to its clinical advantages, such as higher flexibility after insertion into the body and lingered indwell time.

**Figure 1:**
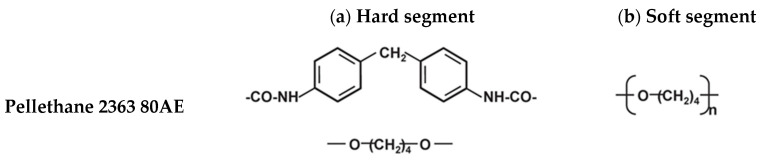
Chemical Structure of Pellethane 2363 80AE. (**a**) Methylene diphenyl isocyanate and 1, 4-butanediol, a chain extender hard segments and (**b**) poly (tetramethylene glycol) soft segments.

Thermoplastic polyurethane (TPU), a family member of PU, has superior mechanical integrity, durability, and resistance against oils and chemicals. The TPUs are composed of alternating block copolymer molecules between rigid and flexible segments. Structurally, TPU is a multi-phase separated block or segmented copolymer which is basically synthesised by three raw materials bonded by a urethane linkage. These three components consist of a polyol or long-chain diol, a chain extender or short-chain diol, and a diisocyanate. Properties of the TPU depend on the molecular weight and ratios of these components. Structurally, the TPU consists of blocks alternating between soft and hard segments forming a two-phase microstructure. Generally, phase separation occurs in most TPUs due to the intrinsic incompatibility between the hard segments and soft segments [6,7]. Furthermore, TPUs can be easily processed by hot melt extrusion, injection moulding, and thermoforming [8]. In this study, Pellethane 2363 80AE, an aromatic polyether-based thermoplastic polyurethane elastomer, was selected. It consists of an aromatic isocyanate, a 4,4-methylenediphenyl as a hard segment, and polyether polyol, a poly(tetramethylene oxide) as a soft segment, as shown in Figure 1 [9]. It offers good flexibility suitable for either injection moulding or extrusion. It is popular for medical applications, such as tubing, catheters, and other short-term uses. It also complies with US Pharmacopoeia XXII Class VI guidelines. The polymeric hot melt extrusion process is one of the primary processes for the manufacturing of medical devices, including catheters.

Hot-melt extrusion (HME) process is typically used in manufacturing such profiles. The HME is defined as a continuous manufacturing process, in which the raw plastic is melted and subsequently formed in a continuous profile along the barrel, before it finally exits the die to solidify using a cooling treatment involving either an air or water bath [10,11]. With this processing technique, a broad range of products, including medical devices, can be fabricated, such as sutures, catheters, and medical tubes. However, one of main hurdles associated with the high-temperature processing often required for HME is that the incorporation of thermosensitive additives or active pharmaceutical ingredients can be restricted for biomedical plastic devices unless the addition of plasticiser is incorporated to lower the viscosity, resulting in a reduction in melt process temperature [12]. Nonetheless, most of the plasticisers which are currently used are chemical agents, which limit their applications in biomedical fields due to their solvent residue. Supercritical fluid (SCF) is an alternative environmentally friendly transient plasticiser which has garnered great interest in facilitating the processing of polymers. The SCF is a substance at which both pressure and temperature are above its critical values, and its properties lie between those of gases and liquids [13]. Several SCFs are being used in the industry, such as carbon dioxide, nitrogen, water, and methanol. Among the SCFs, supercritical carbon dioxide (scCO_2_) is considered as a promising ecological and economical green source, as it is non-toxic, non-flammable, chemically inert, easily recyclable, inexpensive, safe to use, and easily achieves critical states (Tc = 31 °C, Pc = 7.38 MPa). Furthermore, SscCO_2_ acts as a transient plasticiser which can increase the mobility of the polymer chains, resulting in lower viscosity and operational temperature, modify the rheological properties, and enhance the mechanical properties and homogeneity in the case of adding fillers or particles into the polymer base [14,15,16]. In addition, sub- and near-critical CO_2_ considered as milder processing conditions have been widely studied in various polymer applications [17,18,19].

Thus, the combination of HME and SCF technology can open up new dimensions in polymer processing manufacturing. Recently, research on scCO_2_-assisted HME processes has received attention in biomedical and pharmaceutical applications [20,21,22,23,24]. For example, Verreck et al. [25] revealed that the scCO_2_ lowered the melt process temperature of Eudragit E100 in the extruder. In another work from Verreck et al., the scCO_2_ was added into an intermeshing co-rotating twin-screw melt extruder for the manufacture of thermosensitive p-aminosalicylic acid (p-ASA) and ethylcellulose 20 cps (EC 20 cps). The results showed that the processing temperature was decreased from 115 °C to 80 °C, which resulted in less degradation of the p-ASA (5%) compared to the extrudates without scCO_2_ treatment (17%), confirmed by DSC and TGA, respectively.

Therefore, the main aim of this work is to investigate the possibility of decreasing the melt processing temperature of Pellethane TPU in the HME process. In this study, the Pellethane TPU was processed using a co-rotating twin-screw extruder with the aid of pressurised CO_2_ at its sub-critical, 4.14 MPa, and near-critical pressure, 6.89 MPa, respectively. Morphological, thermal, rheological, and mechanical properties of the Pellethane TPU were also investigated in this study. This study could provide a novel method to decrease melt process temperature of Pellethane TPU and obtain non-foam Pellethane extrudates.

## 2. Materials and Methods

### 2.1. Materials

Pellethane grade 2363-80 AE (USP Class VI) was supplied from Lubrizol Advanced Materials, Inc. USA. It is an aromatic polyether based thermoplastic polyurethane. Its specific properties are tabulated in Table 1.

### 2.2. Experimental

**Figure 2:**
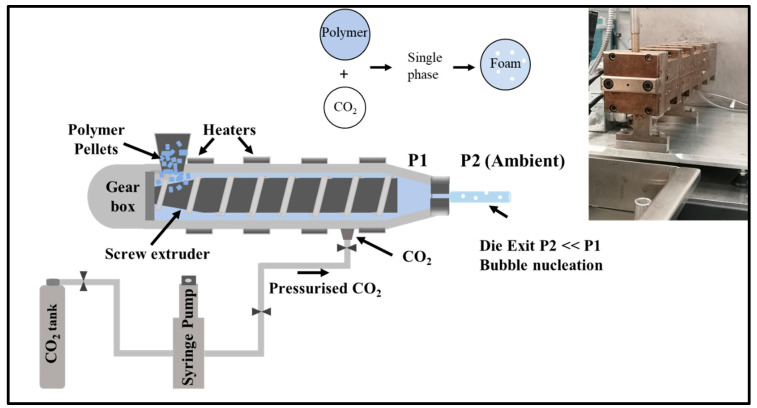
Schematic representation of the pressurised CO_2_-assisted hot melt extrusion setup and a twin-screw extruder used in this experiment (SCF Processing Ltd.) (Right corner).

Pellethane tends to absorb moisture easily due to its hygroscopic nature. The pellets were dried at 72 °C in a circulating air oven overnight prior to processing. Then, HME was carried out on a co-rotating intermeshing twin-screw extruder manufactured by SCF Processing Ltd. The screw had a length to diameter ratio (L/D) of 25:1, and the diameter of the screw was 16 mm. Figure 2 illustrates the schematic diagram of the scCO_2_-assisted co-rotating twin-screw extruder system and an image of the twin-screw extruder used in this study. Briefly, carbon dioxide (CO_2_) was pumped from a cylinder by the 260D high precision syringe pump (Teledyne Isco, Inc. Lincoln, NE, USA). The CO_2_ was then pressurised inside the pump in a constant volumetric flow rate mode, and then the pressurised CO_2_ was introduced at the same pressure as the pressure inducing in the extruder. The injection port was positioned at about 120 mm from the hopper, in a zone where the screw diameter was kept constant. The polymer–CO_2_ blend was then passed and exited through to the die and cooled in a controlled water bath. The pressure, temperature and the volumetric CO_2_ flow rate were measured within the syringe pump. The barrel temperature was carefully regulated at five locations, namely T1, T2 before the CO_2_ injection port, T3 and T4 after the CO_2_ injection port, and T5 before the die, respectively. In this study, three different scenarios, namely extrusion without CO_2_, and with sub- and near-critical CO_2_ at 4.14 and 6.89 MPa, respectively, were evaluated in this study. The samples were first extruded with a filament die and pelletised. After that, the compounded materials were re-extruded with a tape die. The process temperature was lowered for the re-extrusion process, and we obtained non-foam extrudates of the compounded materials from the extrusion with CO_2_ at both 4.14 and 6.89 MPa.

### 2.3. Characterisation

#### 2.3.1. Scanning Electron Microscopy (SEM)

Morphological properties of the Pellethane extrudates were observed using a Hitachi S-2460N scanning electron microscope. Prior to the scanning, the samples were placed on an aluminium holder and sputter-coated with gold under vacuum in an argon atmosphere. The accelerating voltage and filament used were 18 kV and 40–60 mA, respectively.

#### 2.3.2. Throughput Measurement

Throughput measurement was performed during the extrusion process, and the extrudate was measured within a one-minute timeframe in order to evaluate the possible enhancement in terms of productivity in the unit of grams per one minute.

#### 2.3.3. Differential Scanning Calorimetry (DSC)

The DSC studies of the samples were carried out using a DSC Q2000 equipped with a refrigerated cooling system (RCS 90, TA Instruments, New Castle, DE, USA). Samples (approximately 5.0–10.0 mg) were accurately weighed and sealed into aluminium pans. An empty crimped aluminium pan was used as the reference pan. Nitrogen was used as the purging gas at a flow rate of 50 mL/min. The heat/cool/heat experiment was conducted from −80 °C to 250 °C using a heating and cooling rate of 10 °C/min for all the samples. The analysis was carried out using TRIOS Thermal Analysis software, Version 4.5.1.

#### 2.3.4. Thermogravimetric Analysis (TGA)

The TGA experiments were performed with a TGA Q50 thermogravimetric analyser (TA Instruments, USA). The samples were evenly placed in an opened aluminium pan and then placed on the platinum pan of 6.4 mm diameter and 3.2 mm depth, with an initial sample mass of 10–15 mg. Nitrogen gas was used for purging the sample vertically (40 mL/min) and horizontally (60 mL/min). The samples were heated up to 550 °C at a heating rate of 10 °C/min. The analysis was carried out using TRIOS Thermal Analysis software, Version 4.5.1.

#### 2.3.5. Rheological Properties

The rheological properties of the Pellethane were measured using an oscillatory rheometer TA Instruments Discovery Hybrid Rheometer 2 (DHR-2) with a parallel plate geometry of 25 mm diameter and a 1 mm gap to determine the viscosity and viscoelastic properties of the samples. Firstly, the oscillation amplitude test was carried out to identify a strain value within the linear viscoelastic region (LVR). The temperature was set at 185 °C with a frequency of 1 Hz, and % strain ranged from 0.1 to 20%. After that, the oscillation frequency test was conducted by keeping the strain constant at 2%, and the angular frequency was varied between 0.1 and 100 rad/s. The analysis was carried out using TRIOS Rheological Analysis software Version 4.5.1.

#### 2.3.6. Tensile Tests

Tensile testing was conducted on five replicates with a Zwick/Roell model Z010 universal testing machine. For this test, ASTM D412 was applied. A 2.5 kN load cell was used, and the crosshead speed was applied at 500 mm/min. The tensile tests were undertaken at room temperature on rectangular samples with dimensions of approximately 1.5 mm (thickness) and 15 mm (width). The analysis was carried out using testXpert testing software Version II. Tensile modulus at 50%, 100%, and 300% elongation and final strength and elongation values were all calculated.

## 3. Results and Discussion

### 3.1. Effect of CO_2_ on Process Parameters

Table 2 shows the extrusion parameters of Pellethane with and without pressurised CO_2_. The change in viscosity of polymer melt in the barrel can be observed from extrusion torque and die pressure [21]. The recorded torque and die pressure readings shown in Table 2 were slightly decreased from 5 to 3 psi and 16 to 13 N/m, respectively, when the scCO_2_ was injected into the extruder while the process temperature and screw speed were kept constant. A drop in the melt viscosity required less torque and die pressure to push the polymer melt to exit the die. The depression in viscosity was confirmed by rheology testing.

The sorption of CO_2_ molecules into the polymer matrix acting as a lubricant in molecular level caused an increase in free volume and a reduction in chain entanglements, resulting in an increase in the mobility of the polymer chains. This resulted in a reduction in melt viscosity and glass transition temperature [26]. The Pellethane grade employed in this study is a block copolymer which consists of aromatic isocyanate, a 4,4-methylenediphenyl as a hard segment, and polyether polyol, a poly(tetramethylene oxide) as a soft segment as shown in Figure 1 [27]. The accessibility of CO_2_ to the polymer matrix was improved, as it penetrated easier into the polyether polyol chains due to the soft domains and amorphous-like component. In addition, the weak Lewis acid–base interactions between the CO_2_ (electron receptor) and polyether soft segment (electron donor) resulted in sorption and the swelling of the matrix.

In addition, no change in torque was observed when the CO_2_ pressure increased from 4.14 up to 6.89 MPa at the same temperature profile with the untreated Pellethane, while the torque of the Pellethane at 6.89 MPa very slightly decreased compared to 4.14 MPa when reducing the process temperature. Both pressures used in this study were below the supercritical condition of CO_2_ (7.38 MPa). No significant difference in terms of extrusion process was observed. The Pellethane extrudates which exited from the die were foamy, flashing, or discontinuous, and were difficult to collect at the pressure above the supercritical states, such as 8.27 and 10.34 MPa, using the same screw speed, process temperatures, and CO_2_ flow rate.

Regarding the evaluation of pressurised CO_2_ on the process temperature, the lowest melt temperature where it was still processable was 160 °C for the Pellethane both at 4.14 and 6.89 MPa. This melting temperature was decreased by 25 °C as compared to the melt temperature without the assistance of CO_2_ with the theoretical reduction in the glass transition temperature. The aid of CO_2_ in HME could enlarge the processing window in the case of Pellethane by 25 °C. In addition, it could also reduce thermal degradation and energy consumption, increase throughput, and improve the cost-effectiveness of extrusion processing.

### 3.2. Throughput of Pellethane Treated with and without CO_2_

The average throughput readings in a one-minute time frame for Pellethane TPU are summarised in Table 3. Overall, the samples treated with CO_2_ at both 4.14 and 6.89 MPa had a higher average weight compared to the samples treated without CO_2_. This enhancement is due to the plasticisation effect, the lower viscosity of which resulted in a faster flow throughout the extruder, causing an increase in throughput. The addition of CO_2_ enabled an improvement in throughput productivity from 8.33 g for the samples treated without CO_2_ to 10.31 and 10.53 g/min. The throughput increased by 23.77 and 26.41% at 4.14 and 6.89 MPa, respectively. In other words, under the same processing conditions, such as temperature profile, and screw speed, which consumes the same or comparable amount of energy, CO_2_ can help produce a higher mass of extrudates during the given timeframe. Therefore, CO_2_ can be considered as a potential cutting-edge plasticiser in reducing the energy consumption which, hence, leads to greater improved cost-effectiveness during Pellethane TPU processing.

### 3.3. Morphological Characterisation

**Figure 3:**
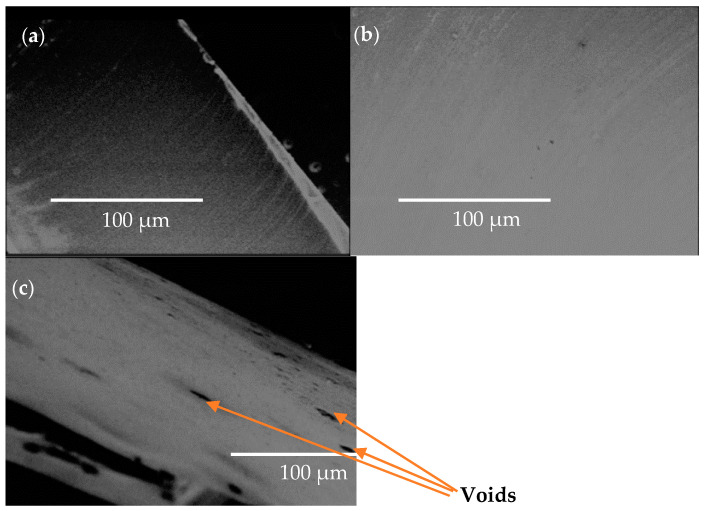
SEM images of Pellethane TPU (**a**) without CO_2_ (control), (**b**) with CO_2_ at 4.14 MPa, and (**c**) with CO_2_ at 6.89 MPa (images captured at x400, 18 kV, and 100 μm).

SEM photographs of Pellethane TPU samples after re-extrusion are shown in Figure 3. The results revealed that the porosity was not observed from the Pellethane sample treated with CO_2_ at 4.14 MPa. Its surface morphology was similar to the SEM image of the virgin Pellethane TPU.

Interestingly, voids were observed of approximately 10–20 μm on the surface of the Pellethane TPU sample treated with near-critical scCO_2_ at 6.89 MPa. This was probably because it was at near-scCO_2_ where more foamy products were obtained. Such a structure did not decrease the mechanical properties of Pellethane TPU. Probably, the growth and rearrangement of the crystal domains had a greater effect on the mechanical performance of the Pellethane TPU.

### 3.4. Rheological Properties

**Figure 4:**
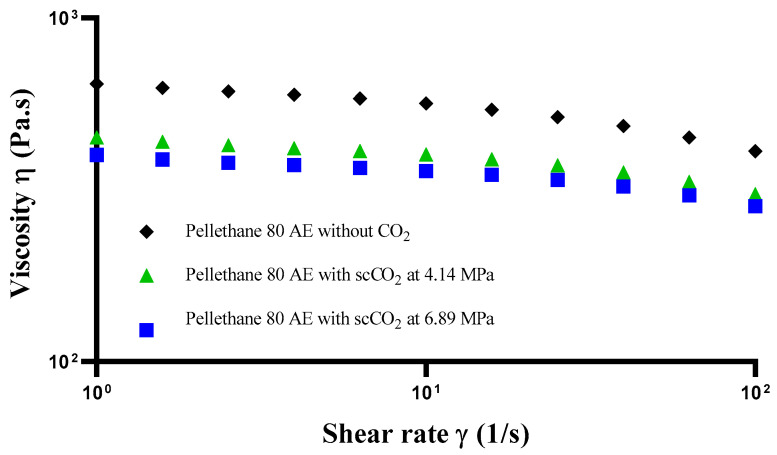
Viscosity dependence on shear rates for three different scenarios.

To ascertain the changes in the rheological properties of Pellethane which occurred in the barrel in the presence of pressurised CO_2_ at both 4.14 and 6.89 MPa, a rotational rheometer coupled with 25 mm parallel plates was employed. The samples were heated at 185 °C, and rheological analysis was performed using the dynamic frequency sweep mode to identify their linear viscoelastic region (LVR) region. Once the LVR was determined, the complex viscosity data in the range of LVR was then transformed by applying the Cox–Merz rule [28]. Figure 4 shows the changes in viscosity of the during different scenarios as a function of shear rate.

Overall, the viscosity of the samples decreased with increasing shear rate. Clearly, the viscosity of the samples treated with pressurised CO_2_ at both 4.14 MPa and 6.89 MPa was lower than the sample without the addition of pressurised CO_2_ over the shear rate between 0.1 and 100 1/s. However, no significant difference was found between the pressurised CO_2_ at 4.14 and 6.89 MPa. The experimental viscosity data were then fitted to the Carreau model [29], which was used to explain rheological behaviour, as shown Equation (1) in order to enable quantitative determination of the zero-shear viscosity, as shown in Table 4. The model is given by as follows:

(1) $$\frac{\eta -\eta_{\infty }}{\eta_{0}-\eta_{\infty }}=\frac{1}{{\left(1+{\left(k\dot{\gamma }\right)}^{2}\right)}^{\frac{n}{2}}}$$

where η_o_ is the Newtonian viscosity, η_∞_ is the infinite viscosity, γ is the effective shear rate, *k* is the consistency, and *n* is the power-law index +1.

This provided a rheological viewpoint to the CO_2_-induced T_g_ depression of this polymer which correlated well with the DSC results and the operating temperature within the extruder.

### 3.5. DSC

In order to evaluate if the effect of pressurised CO_2_ during the extrusion processing of the Pellethane matrices had any lingering effect on their thermal behaviours, the melting temperature (T_m_), glass transition temperature (T_g_), and crystallisation temperature (T_c_) were assessed by DSC. The results are summarised in Table 5. At the first heating, the cycle was used to delete the thermal history. Therefore, the second heat cycle was then employed to determine T_g_ and T_m_ in this study. Clearly, the CO_2_ affected the depression of T_g_, as it decreased by approximately 8 °C compared to the T_g_ value of the neat Pellethane. The sorption of CO_2_ into a polymer was able to act as a plasticiser, resulting in an increase in the mobility of the polymeric chains specifically in the amorphous regions which was evidenced by the reduction in the T_g_ of the polymer, which in turn resulted in a drop in the melt viscosity, as mentioned previously [16]. As for T_m_, no significant changes were found among the three different samples. Interestingly, a significant change in T_c_ of the Pellethane treated with CO_2_ at 6.89 MPa was observed. It was 75.18 °C, while the T_c_ of the virgin Pellethane and Pellethane treated with CO_2_ at 4.14 MPa was about 128.89 °C. The suggested reason behind this substantial change was that, at 6.89 MPa, the higher increase in the rearrangement of the crystalline phase then resulted in it taking more time or a lower temperature to re-crystallise. The scCO_2_ could not only disrupt an amorphous phase but also a partial or total part of the crystalline phase of the semi-crystalline polymer [30,31].

### 3.6. Thermal Stability

**Figure 5:**
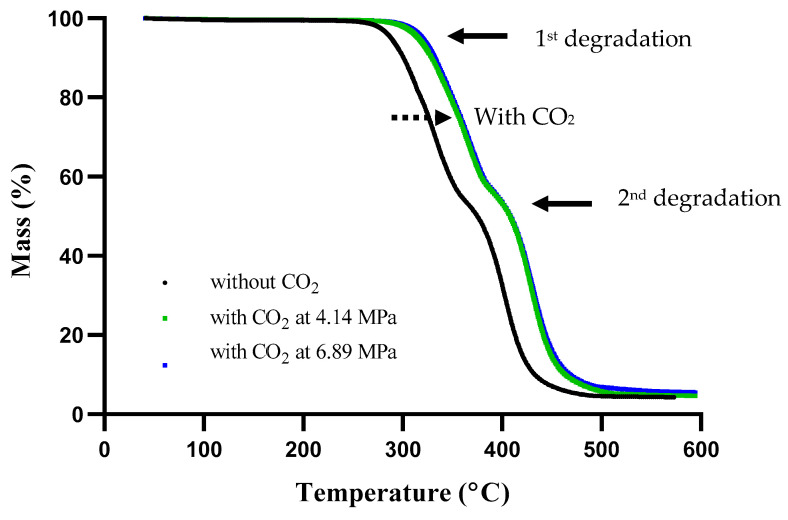
TGA thermogram of Pellethane TPU with and without CO_2_.

The thermal degradation profiles of Pellethane TPU extrudates were obtained from TGA. Two major steps were clearly observed in the Pellethane thermal degradation at all scenarios, as represented in Figure 5.

The first degradation step occurred at a temperature range from 280 to 380 °C, which was related to the degradation of hard segments due to the breakdown of the urethane linkages, whereas the second degradation step which took place between 380 and 520 °C was associated with the decomposition of soft segments, polyols into hydrocarbons, and oxygenated moieties [32]. In addition, the shift in degradation profile was observed in the samples extruded with the assistance of CO_2_ at both 4.14 and 6.89 MPa. A slight increase in the degradation temperature was possibly due to the rearrangement of crystalline chains in the hard domains, which made it more stable compared to the sample treated without CO_2_. However, no difference between the sample treated at 4.14 and 6.89 MPa was observed.

### 3.7. Mechanical Properties

**Figure 6:**
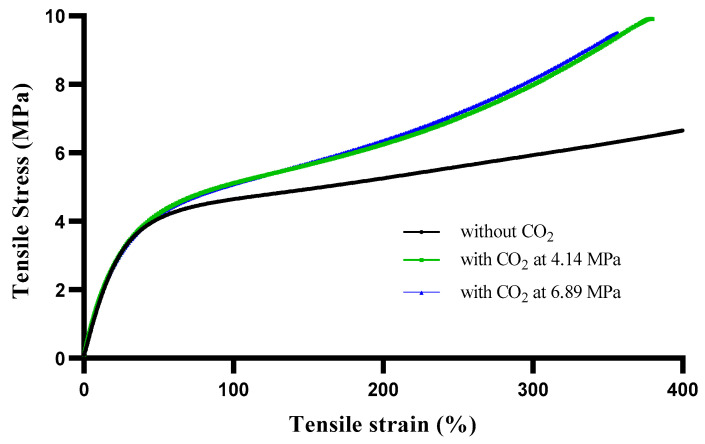
Stress–strain relationship of Pellethane TPU with and without CO_2_.

In addition to thermal characterisation, this study also examined the effect of pressurised CO_2_ on the possible changes in mechanical performance of Pellethane TPU. Figure 6 illustrates the transition from rigid plastic to the elastomeric behaviour of Pellethane TPU samples with and without CO_2_. The results showed that the tensile modulus at 50% elongation amongst three different scenarios was not significantly different, but that the tensile modulus at 100% and 300% and also the final strength and elongation increased in the samples treated with CO_2_, as shown in Table 6. The enhancement in the elongation and toughness of Pellethane TPU in the presence of CO_2_ was due to the increased volume of crystallinity and the rearrangement of the Pellethane TPU microphase, allowing the structure to behave in a more kinetically favourable manner, as reported by Karode et al. [33]. The results indicated that Pellethane TPU treated with CO_2_ absorbs energy under greater loads and elongation compared to untreated Pellethane TPU. However, increasing the pressure from 4.14 to 6.89 MPa did not cause significant macroscopic changes to the mechanical properties of Pellethane TPU.

## 4. Conclusions

Pellethane TPU was extruded using sub- and near-critical CO_2_ to investigate its morphological, thermal, rheological, and mechanical properties. The sorption of CO_2_ at both 4.14 and 6.89 MPa into Pellethane TPU increased the mobility of the polymeric chains, especially in the soft domain, which were evidenced by the reduction in the T_g_, T_c_, and viscosity. In addition, the throughput, thermal stability, and mechanical properties were also enhanced. Thus, the use of near-critical and sub-critical CO_2_ as processing aids for HME provides a novel method to successfully depress the melt process temperature of Pellethane TPU from 185 °C to 160 °C. This enlarged the window for temperature processing by 25 °C or possibly wider for other polymers, and opens up opportunities for incorporating thermally labile drugs to withstand the molten state of Pellethane polyurethane used in medical catheter manufacturing.

## Figures and Tables

**Table 1:** Specific properties of Pellethane 2363 80AE.

Properties	Test Method	Values
Shore hardness	ASTM D 2240	85-A
Glass transition temperature (T_g_)	DSC	−47 °C
Vicat softening temperature	ASTM D 1525	81.7 °C
Melt flow rate, 190 ºC, 8700 g, g/10 min	ASTM D 1238	10
Tensile modulus 50% elongation, MPa 100% elongation, MPa 300% elongation, MPa	ASTM D412	4.8 MPa 6.1 MPa 10.3 MPa
Ultimate tensile strength, MPa	ASTM D 412	28.9 MPa

**Table 2:** Extrusion conditions and processing observations of Pellethane TPU with and without CO_2_.

Process Parameters	(1) Extrusion without CO_2_ (Control)	(2) Extrusion with CO_2_ @4.14 MPa	(3) Re-Extrusion with the Compounded Material from (2)	(4) Extrusion with CO_2_ @6.89 MPa	(5) Re-Extrusion with the Compounded Material from (4)
Temperature at zone 1 (°C)	114	116	103	114	103
Temperature at zone 2 (°C)	170	170	150	170	150
Temperature at zone 3 (°C)	180	180	155	180	155
Temperature at zone 4 (°C)	185	185	160	185	160
Temperature at melt (°C)	188	186	163	186	162
Temperature at die (°C)	185	185	160	185	160
Extruder torque (N/m)	16	13	15	13	14
Die pressure (MPa)	2.76	1.65	2.21	1.65	2.21
Screw speed (rpm)	55	55	60	55	60
CO_2_ pressure (MPa)	-	4.14	-	6.89	-
CO_2_ flow rate (ml/min)	-	0.5	-	0.5	-
CO_2_ temperature (°C)	-	35	-	35	-

**Table 3:** Average weights of Pellethane TPU extrudates treated with and without CO_2_ in a one-minute time frame.

Scenario	Average Weight of Extrudate (g)	% Increase
Without CO_2_ (control)	8.33 ± 0.10	-
With CO_2_ at 4.14 MPa	10.31 ± 0.06	23.77%
With CO_2_ at 6.89 MPa	10.53 ± 0.07	26.41%

Results are mean ± standard deviation, *n* = 5.

**Table 4:** Zero-shear viscosity of Pellethane at three different scenarios.

Scenario	Zero-Shear Viscosity (Pa.s)	Regression (R^2^)	% Reduction
Without CO_2_ (control)	690.407	0.9995	-
With CO_2_ at 4.14 MPa	439.374	0.9996	36.36%
With CO_2_ at 6.89 MPa	413.900	0.9953	40.04%

**Table 5:** DSC summary results at different scenarios.

Sample	T_g_ (°C)	T_m_ (°C)	Enthalpy (J/g)	T_c_ (°C)	Enthalpy (J/g)
Without CO_2_ (control)	−56.07	167.27	3.97	128.89	5.74
With CO_2_ at 4.14 MPa	−61.65	161.16	2.37	76.79	4.74
With CO_2_ at 6.89 MPa	−64.93	155.37	2.25	75.18	4.79

**Table 6:** Mechanical properties of Pellethane TPU with and without CO_2_.

Scenario	Tensile Modulus 50% Strain (%)	Tensile Modulus 100% Strain (%)	Tensile Modulus 300% Strain (%)	Final Tensile Strength (MPa)	Final Strain (%)
Without CO_2_ (control)	4.08 ± 0.82	4.65 ± 0.30	5.93 ± 0.27	6.65 ± 0.39	415.25 ± 20.00
With CO_2_ at 4.14 MPa	4.09 ± 0.41	5.00 ± 0.50	7.71 ± 0.85	10.08 ± 0.91	428.72 ± 27.45
With CO_2_ at 6.89 MPa	4.15 ± 0.19	5.05 ± 0.24	7.71 ± 0.41	10.20 ± 0.86	422.86 ± 21.02

Results are mean ± standard deviation, *n* = 7.

## Data Availability

Data available on request due to restrictions eg privacy or ethical.
